# Building Perinatal Pathology Research Capacity in Sub-Saharan Africa

**DOI:** 10.3389/fmed.2022.958840

**Published:** 2022-07-08

**Authors:** Lisa M. Bebell, Joseph Ngonzi, Frederick A. Meier, Chrystalle Katte Carreon, Abraham Birungi, Vanessa B. Kerry, Raymond Atwine, Drucilla J. Roberts

**Affiliations:** ^1^Division of Infectious Diseases, Department of Medicine, Medical Practice Evaluation Center and Center for Global Health, Massachusetts General Hospital, Harvard Medical School, Boston, MA, United States; ^2^Department of Obstetrics and Gynecology, Faculty of Medicine, Mbarara University of Science and Technology, Mbarara, Uganda; ^3^Department of Pathology, Wayne State University School of Medicine, Detroit, MI, United States; ^4^Division of Women’s and Perinatal Pathology, Department of Pathology, Brigham and Women’s Hospital, Boston, MA, United States; ^5^Department of Pathology, Boston Children’s Hospital, Harvard Medical School, Boston, MA, United States; ^6^Department of Pathology, Faculty of Medicine, Mbarara University of Science and Technology, Mbarara, Uganda; ^7^Division of Pulmonary and Critical Care Medicine and Center for Global Health, Department of Medicine, Massachusetts General Hospital, Boston, MA, United States; ^8^Harvard Medical School, Seed Global Health, Boston, MA, United States; ^9^Department of Pathology, Massachusetts General Hospital, Harvard Medical School, Boston, MA, United States

**Keywords:** Uganda, placenta, fetus, histopathology, histology, outcomes, pregnancy, stillbirth

## Abstract

**Introduction:**

Over two million stillbirths and neonatal deaths occur in sub-Saharan Africa (sSA) annually. Despite multilateral efforts, reducing perinatal mortality has been slow. Although targeted pathologic investigation can often determine the cause of perinatal death, in resource-limited settings, stillbirths, early neonatal deaths, and placentas are rarely examined pathologically. However, the placenta is a key source of diagnostic information and is the main determinant of fetal growth and development *in utero*, influencing child health outcomes.

**Methods:**

In 2016, our collaborative intercontinental group began investigating infectious perinatal death and adverse child health outcomes in Uganda. We developed and initiated a 4-day combined didactic/practical curriculum to train health workers in placental collection, gross placental examination, and tissue sampling for histology. We also trained a local technician to perform immunohistochemistry staining.

**Results:**

Overall, we trained 12 health workers who performed gross placental assessment for > 1,000 placentas, obtaining > 5,000 formalin-fixed tissue samples for research diagnostic use. Median placental weights ranged from 425 to 456 g, and 33.3% of placentas were < 10th percentile in weight, corrected for gestational age. Acute chorioamnionitis (32.3%) and maternal vascular malperfusion (25.4%) were common diagnoses.

**Discussion:**

Through a targeted training program, we built capacity at a university-affiliated hospital in sSA to independently perform placental collection, gross pathologic examination, and placental tissue processing for histology and special stains. Our training model can be applied to other collaborative research endeavors in diverse resource-limited settings to improve research and clinical capacity and competency for diagnostics and management of stillbirth, neonatal death, and child health outcomes.

## Introduction

There are at least 5.5 million stillbirths and neonatal deaths annually (1), and > 40% occur in sub-Saharan Africa (sSA). Despite multilateral efforts, reducing global perinatal morbidity and mortality has been slow, especially in sSA (2). The placenta is the main determinant of fetal growth and development *in utero* (3) and mediates maternal adaptations and maladaptions to pregnancy (4–6), reflecting or influencing a multitude of fetal and child health outcomes (7). One poor outcome is fetal growth restriction. Fetal growth restriction is diagnosed when the fetus fails to meet its full growth potential due to pathological factors *in utero*. Growth restriction most often results from placental dysfunction (8). Globally, fetal growth restriction is a leading cause of stillbirth, neonatal mortality, and childhood and long-term morbidity (8). For these reasons, the International Federation of Gynecology and Obstetrics (FIGO) strongly recommends sending placentas from pregnancies with fetal growth restriction for histopathological examination, and reporting results according to the Amsterdam consensus diagnostic nosology (9). The FIGO recommendation recognizes that placental examination improves diagnostic precision and that the information it provides is often useful when counseling women about the cause of adverse pregnancy outcomes or other obstetric events and for providing guidance on future pregnancies, particularly on the risk of known recurrent placental disorders (8). Furthermore, pathological data on causes of stillbirth, fetal growth restriction, and other undesirable outcomes are necessary to develop appropriate public health initiatives at all levels, plan for clinical services including primary and referral needs, that ultimately will help achieve maternal and child health goals (10, 11).

Unfortunately, though targeted investigation can determine the cause of perinatal death in most cases, stillbirths and early neonatal deaths are rarely investigated using fetal or neonatal autopsy or placental pathology in resource-limited settings (12). Lack of pathology capacity is the main reason placental and fetal examination is rarely performed in sSA. Lack of capacity is multifactorial, including a scarcity of institutions capable of providing training in pathology techniques and research methods and an alarming scarcity of trained pathologists (10, 11). A 2012 survey of pathology resources in sSA demonstrated that all countries except South Africa and Botswana had fewer than one pathologist for every 500,000 people, less than 10% of the pathologist availability in the United States of America (11). Since the 1990s, we have been working in resource-limited settings to build capacity for perinatal pathology and carry out research studies to determine causes of maternal, fetal, and child morbidity and mortality (13–16). Through our individual and collective experience, we have witnessed the dire need to further improve the capacity for placental pathology in sSA.

Here, we report on our program’s experience building perinatal pathology capacity at Mbarara University of Science and Technology (MUST) and its affiliated Mbarara Regional Referral Hospital in southwestern Uganda. Recognizing that human resources for pathology training and research are limited in this setting, as elsewhere in sSA, we leveraged a 20-year multilateral research and teaching partnership involving MUST, Massachusetts General Hospital (MGH), and, more recently, Seed Global Health, to train a cadre of health workers and laboratory technicians to perform gross and histologic placental examination. We detail the structure of the training program and its outcome including quality of prepared slides and identified pathologic placental diagnoses.

## Methods

In 2016, our collaborative intercontinental research group comprised of investigators and educators affiliated with MUST and MGH began a research program investigating infectious causes of stillbirth, early neonatal death, and adverse child health outcomes in Uganda. One team member (FAM), a perinatal pathologist from the United States with specialized training and extensive perinatal pathology experience, was stationed full-time on site at MUST for over 2 years, a volunteer position facilitated by the Seed Global Health program. Seed is a not-for-profit organization focused on human resource for health capacity-building in sSA through sustained collaborative engagement.^1^ Another team member (LMB), an infectious diseases physician and intensivist trained in the United States, lived on-site for 3 years coordinating the program’s development and carrying out research projects with a local obstetrician collaborator (JN). A third team member (DJR, an experienced perinatal pathologist) traveled to the site several times per year to provide the on-site team with support from the MGH Pathology Department and assist with building program capacity, especially training of a local team member (AB) in immunohistochemistry techniques.

Before initiating this program, placentas in Mbarara were rarely examined at birth for either pathologic gross or histologic changes. We began our initiative by developing a training program in gross placental examination and sampling for histologic review, starting with a 4-day combined didactic/practical curriculum to train nurses, midwives, and junior physicians in placental collection and gross assessment to identify lesions that would need sampling for histology. An example training schedule is provided in Table 1, which consisted of combined didactic presentations on placental function and structure and hands-on training in gross placental examination, umbilical cord blood sampling, placental dissection, and sampling of placental disc, membranes, and umbilical cord, and formalin-fixation of samples and sample trimming for histopathology block creation. Often, this training program was embedded in a larger training curriculum on research ethics and principles of informed consent along with additional procedures and data collection relevant to placental and maternal-child research. We employed a train-the-trainer model, with two experienced perinatal pathologists (DJR, FAM) who trained junior pathologists (including RA) and one junior physician scientist (LMB), who further trained local health workers. After carrying out placental gross examination and sampling procedures for at least 2 months, local health workers were then capable of training peers. The training program was modified during the COVID-19 pandemic to include remote live video didactic sessions (conducted by LMB) paired with in-person peer training (conducted by prior trainees with > 2 months of hands-on experience). One author (DJR) also separately facilitated training a histology technician (AB) to perform immunohistochemistry. This focused training program consisted of methods to prepare tissue for immunohistochemistry, battery testing, quality control and assurance practices, antigen retrieval, direct and indirect methods of manual immunohistochemistry staining, and development of a standardized operating protocol for immunohistochemistry.

**TABLE 1:** Training program in gross placental examination and sampling for health workers.

Day (total time)	Training activity	Type of activity	Participants and roles	Activity duration
Day 1 (4–6 h)	Introduction to placental anatomy, physiology, and pathology	Didactic (computer slide-based or video lecture)	Trainer-led talk attended by trainees	45 min
	Overview of placental procedures: • Gross placental examination • Sampling umbilical cord blood • Gross placental pathology • Sampling placental disc, membranes, umbilical cord • Formalin fixation and trimming	Didactic (computer slide-based or video lecture)	Trainer-led talk attended by trainees	1 h
	Demonstration of gross placental examination and sampling	Hands-on practical	Trainer-led, attended by trainees	2 h
	Practice gross placental examination and sampling	Hands-on practical	Trainees, observed and coached by trainer	1–2 h
Day 2 (3–5 h)	Demonstration of placental specimen trimming	Hands-on practical	Trainer-led, attended by trainees	45 min
	Practice placental specimen trimming	Hands-on practical	Trainees, observed and coached by trainer	1–2 h
	Practice gross placental examination and sampling	Hands-on practical	Trainees, observed and coached by trainer	1–2 h
Day 3 (2–4 h)	Practice gross placental examination and sampling	Hands-on practical	Trainees, observed and coached by trainer	2–4 h
Day 4 (3–5 h)	Practice gross placental examination and sampling	Hands-on practical	Trainees, observed and coached by trainer	2–4 h
	Practice placental specimen trimming	Hands-on practical	Trainees, observed and coached by trainer	1–2 h

Research grant funding (to LMB) and a volunteer stipend (to FAM) paid for time spent training health workers in gross placental examination and sampling. Histopathologic and histopathology supplies were funded by research grant funding (to LMB) as well as departmental partnership funds at MGH (to DJR and others). Gross placental examination findings were recorded on a standardized case report form developed by DJR in accordance with her practice and training at Harvard Medical School teaching hospitals. Histopathology findings were recorded on standardized case report forms using the categories defined in the Amsterdam consensus diagnostic nosology (9). Case report forms were then abstracted into a Research Electronic Capture (REDCap) database (17) for analysis.

All research participants provided written informed consent to participate in each research study. The first research study was approved by MUST (12/11-15), Mbale Regional Referral Hospital Research Ethics Committee (082/2016), Partners Healthcare (2016P000806), and Pennsylvania State University College of Medicine (STUDY0004199). The second research study was approved by MUST (11/03-17) and Partners Healthcare (2017P001319). The third research study was approved by MUST (10/06-19) and Partners Healthcare (2019P003248).

## Results

### Placental Gross Assessment and Sampling

**FIGURE 1:**
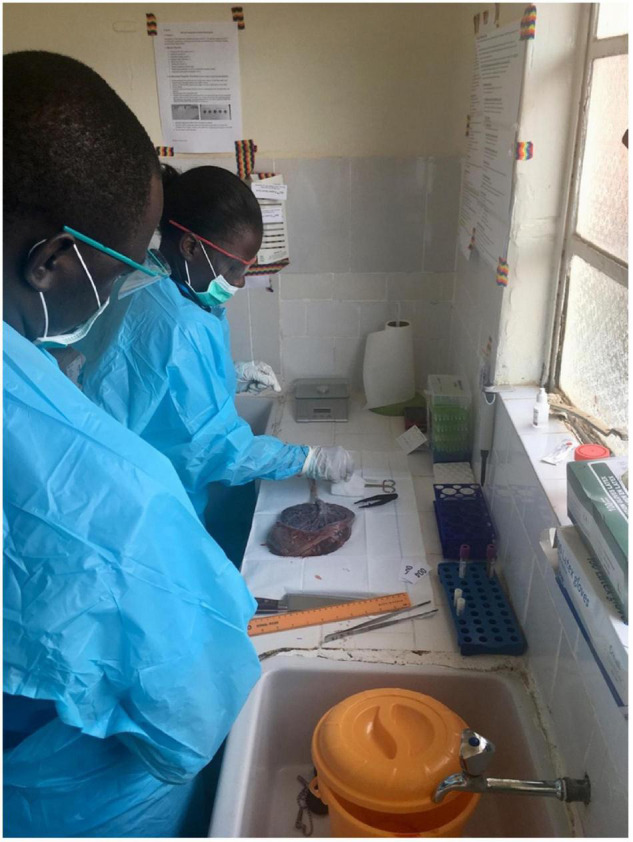
Trained health workers independently dissecting and sampling placenta after gross examination in Mbarara, Uganda.

Training was conducted for three distinct studies over a 5-year time span from 2016 to 2021 (Table 2). One training program per study period was deemed sufficient to train all staff, with the exception of Study 3, when training was carried out in stages to accommodate newly hired staff. Refresher training was also provided due to interruptions in the research program from the COVID-19 pandemic. Overall, we trained 12 health workers, including six midwives, four nurses, and two junior medical doctors. All trainees are now capable of independently performing gross placental examination and collecting samples for histopathologic examination for research and/or clinical purposes (Figure 1). Altogether, the Mbarara trainees have collected, and performed gross placental assessment, on over 1,000 placentas to date (Table 2). Approximately 80% of all histologic slides prepared from these placentas were of adequate quality for diagnostic interpretation. Median placental weights ranged from a mean of 425 grams in Study 1 to a median of 456 (IQR 382–529) grams in Study 2 and 443 (IQR 375–511) grams in Study 3 (Table 2). Overall, 33.3% of placentas were in the < 10th percentile in weight, corrected for gestational age using a standard weight chart (18).

**TABLE 2:** Health workers trained, number of placentas processed and evaluated, and gross and histologic pathology findings in three separate research studies that constitute the foundation of perinatal pathology research at a Ugandan regional referral hospital.

Research project Participants	Project 1 *n* = 100	Project 2 *n* = 352	Project 3* *n* = 600
Years carried out	2016–2017	2017–2018	2019 –
Number of newly trained health workers	4	2	6
Health worker qualifications	Junior medical doctor (1) Nurse (2) Midwife (1)	Junior medical doctor (1) Midwife (1)	Nurse (2) Midwife (4)
Number of returning, previously trained health workers	–	2	3
Placentas evaluated, *n* (%)			
Gross examination	100 (100)	352 (100)	525 (87.5)
Histopathology	100 (100)	352 (200)	118 (19.7)
Number of placental parenchymal histopathology slides per participant case, median (IQR)	2 (2–3)	2 (2–3)	4 (4–4)
“Adequate” histopathology quality, *n* (%)	100 (100)	316 (89.8)	87 (73.7)
Immunohistochemistry (IHC) performed, *n* (%)	0	51 (14.5)	0
Placental weight in grams, median (IQR)	Mean 425	456 (382–529)	443 (375–511)
Placentas < 10th percentile of expected weight for gestational age (*n* = 616)		205 (33.3)	
Histopathology findings			
Acute chorioamnionitis (*n* = 561)		184 (32.3)	
Maternal vascular malperfusion (*n* = 453)		115 (25.4)	

*Study still in progress. Not all data for all studies were available to be included.

### Placental Histopathology

Trainees fixed and trimmed formalin-fixed samples of umbilical cord, membranes, placental parenchyma, and any focal disc or umbilical cord lesions to create formalin-preserved tissue specimens for paraffin embedding. In total, 5,408 tissue samples were processed and used for research diagnostics through the three studies. Grant funding supported local histology technicians (AB and four others) to create paraffin-embedded blocks, hematoxylin and eosin (H&E)-stain slides and generate slides for routine histopathology. Due to human resource constraints on local pathologists, slides were interpreted by perinatal pathologists based in the United States (DJR, FAM, and CKC) according to the Amsterdam consensus diagnostic nosology (9). Common histologic diagnoses included acute chorioamnionitis (present in 32.3% of cases), and features of maternal vascular malperfusion (MVM, present in 25.4% of cases).

### Publications and Other Deliverables

To date, we have published three manuscripts reporting the results of this work (14, 15, 19), with one more currently under review and another three manuscripts in preparation. The local technician (AB) trained to perform immunohistochemistry staining on placental tissue for research (14) and clinical purposes has leveraged these skills to perform immunohistochemistry staining to assist in diagnosis of breast and other cancers.

## Discussion

Through a structured and targeted training program, we built capacity at a university-affiliated hospital in sSA to independently perform placental collection, gross pathologic examination and placental tissue processing for histology and special stains for placentas of diagnostic interest, adequate for research purposes. The health workers trained came from a variety of medical specialties including general nursing, midwifery, pediatrics, obstetrics and gynecology, and pathology. The training program demonstrated that health workers with diverse backgrounds can learn and teach essential placental gross examination and sampling techniques for histopathology. During the social disruption of the early COVID-19 pandemic, we hybridized our training model to include a virtual component that can be applied to other collaborative research endeavors in different resource-limited settings. We advocate for the *train-the-trainer* model to ensure ongoing local development of trainee skills to carry out research, improve on-site clinical diagnostic capacity and patient care, and allow trainees subsequently assume trainer roles to train others going forward.

We found a high proportion (33.3%) of placentas that weighed < 10th percentile of expected weight for gestational age. However, gestational age correction was performed using a standard weight chart based on women in the northeastern United States of America (18) since Ugandan or sSA-specific placental weight charts are not currently available. We believe that placental weights in Uganda appear smaller for gestational age when high-income reference standards are used, likely due to the high percentage of small placentas by weight. Furthermore, measurements aggregated from seven studies in high-resource settings reported a median weight of 520 (10–90% range 408–642) g (20). Thus, gestational age correction using non-local standards may lead to overdiagnosis of low placental weight. However, low placental weight could also indicate placental hypoplasia, a feature of MVM. Combined with small placental size, a relatively high prevalence MVM (25.4%) may point to a relatively high incidence of other features of MVM, including placental infarcts, increased syncytial knots, distal villous hypoplasia, increased perivillous fibrin, etc. Together, these findings are concerning indicators of possible intrauterine fetal growth restriction, which is a leading global cause of stillbirth, neonatal mortality, and morbidity (8). Furthermore, maternal HIV infection and antiretroviral treatment are associated with MVM, with prevalence as high as 30–40% (21). Large-scale perinatal pathology studies carried out in sSA have also demonstrated associations between utero-placental vascular pathology, acute chorioamnionitis, and stillbirth (22, 23). These results, reflecting a miniscule proportion of all deliveries in sSA, highlight the need for histopathological examination of placentas of unexplained stillbirth deliveries, fetal growth restriction, and maternal HIV infection, and the need to use standardized criteria for reporting diagnoses in order to compile and compare results across various settings (22). Alongside developing local placental weight charts, the high prevalence of low placental weight should be further investigated and addressed to improve mortality in children under age five.

Though largely successful, our initiative has several limitations. Only approximately 80% of the slides produced by our trainees were adequate for histopathologic interpretation. The 20% that were inadequate often had issues with processing and fixation but did not have issues with trimming/cutting of specimen tissue or paraffin blocks, or staining. The large proportion of inadequate slides emphasizes the continuing challenge of consistent slide production in resource-constrained settings. Furthermore, although local pathologists based at MUST and its affiliated regional referral hospital are capable of interpreting H&E-stained placental slides, unfortunately, human resource limitations currently limit their capacity to add placental cases to their current practice and prioritize these cases over other important diagnostic applications (e.g., new tumor diagnosis). In our future work, we aim to help define priority clinical cases for placental analysis and build capacity to perform on-site fetal autopsy. In addition, although we trained one histopathology technician to perform immunohistochemistry on placental specimens and other tissues, there remains limited local capacity to routinely perform special stains due to a lack of supplies and funding. However, the acquired immunohistochemistry skills can be readily useful for future research endeavors and clinical projects when supplies are available. Additionally, valuable skills may be passed on to other trainees thereby augmenting the pool of skilled individuals able to perform such tasks.

In the future, we hope that increasing research capacity will also translate into greater use of perinatal pathology for clinical diagnosis and management of stillbirth, neonatal death, and child health outcomes in sSA. Information gained from autopsies of stillbirths and neonatal deaths is essential to designing interventions to improve outcomes. Minimally invasive approaches should be considered as alternatives to conventional autopsy, which are proven acceptable alternatives to conventional pediatric autopsy in resource-limited settings (24). Toward this end, building perinatal pathology research capacity also contributes skills and resources that can have a positive collateral impact on patient care. Some strategies to address the shortage of pathology services include concentrating specialists in tertiary care centers and establishing structured referral systems to improve access. Digital telepathology has also been proposed as a stopgap measure to address the pathologist shortage in sSA, with some success, largely when embedded within long-term international collaborations (25). Multinational partnerships can also help with human resource limitations by providing training and diagnostic consultation, including virtual training, which has proven successful in some settings (26). A first step would be to survey current capacity and develop a tool for assessing needs. Although we report our experience of limited capacity to carry out perinatal pathology service in sSA, the true capacity is currently unclear. Recent efforts to assess anatomic pathology capacity have largely been focused on cancer diagnosis. Thus, regional surveys of perinatal pathology capacity should also be implemented.

Collaborative international research programs can, and should, contribute to clinical and research pathology capacity development in sSA (27). There are several good examples of successful programs in addition to ours, including a pathology program at Anokye Teaching Hospital that became self-sustaining after an 11-year partnership with University Hospital of North Norway and could serve as a model for others. The Anokye Pathology Department now provides surgical pathology, cytology, immunohistochemistry, frozen section services, and residency training, fully independent from international assistance (28). A similar partnership between the Fred Hutchinson Cancer Research Center at the University of Washington and Makerere University and its associated Mulago Hospital led to the establishment of a clinical pathology laboratory at the Uganda Cancer Institute that handled 5,700 tissue diagnoses in 2019 and routinely offers immunohistochemistry services (29). Though these examples are in the cancer field, similar programs could, and should, be established for clinical placental pathology and fetal autopsy. In settings where human resources are especially limited, the ability to conduct a thoughtful autopsy and having a keen eye for identifying gross placental lesions are key elements of perinatal pathology, and training health workers to develop these skills should be prioritized. In settings where histopathology is not available, even weights, measurements, and a thorough gross examination can be critically important, and on occasions sufficient to identify the potential cause of fetal or neonatal death (10).

In conclusion, we provide one example of a successful perinatal pathology research program in sSA that could serve as a model for others, increasing perinatal pathology capacity for both clinical and research applications. Furthermore, we will strive to continue our collaborative partnership for many years to come, building further capacity in clinical diagnostics to improve pregnancy and child health outcomes.

## Data Availability Statement

The raw data supporting the conclusions of this article will be made available by the authors, without undue reservation.

## Ethics Statement

The studies involving human participants were reviewed and approved by the Mbarara University of Science and Technology Institutional Review Committee, Mbale Regional Referral Hospital Research Ethics Committee, Partners Healthcare, and Pennsylvania State University College of Medicine Research Ethics Committee. The patients/participants provided their written informed consent to participate in this study.

## Author Contributions

LB: conceptualization, data curation, formal analysis, funding acquisition, investigation, methodology, project administration, resources, software, supervision, validation, writing—original draft, and writing—review and editing. JN: conceptualization, data curation, investigation, methodology, project administration, resources, supervision, validation, and writing—review and editing. FM: conceptualization, data curation, histopathology slide interpretation, formal analysis, investigation, methodology, project administration, supervision, and writing—review and editing. CKC: conceptualization, data curation, histopathology slide interpretation, supervision, and writing—review and editing. AB and RA: data curation, methodology, project administration, supervision, validation, and writing—review and editing. VK: conceptualization, supervision, and writing—review and editing. DR: conceptualization, data curation, histopathology slide interpretation, formal analysis, investigation, methodology, project administration, resources, supervision, validation, and writing—review and editing. All authors agreed to be accountable for the content of the work.

## Author Disclaimer

The content is solely the responsibility of the authors and does not necessarily represent the official views of Harvard Catalyst, Harvard University and its affiliated academic healthcare centers, the National Institutes of Health, or other funders.

## Conflict of Interest

The authors declare that the research was conducted in the absence of any commercial or financial relationships that could be construed as a potential conflict of interest.

## Publisher’s Note

All claims expressed in this article are solely those of the authors and do not necessarily represent those of their affiliated organizations, or those of the publisher, the editors and the reviewers. Any product that may be evaluated in this article, or claim that may be made by its manufacturer, is not guaranteed or endorsed by the publisher.

## Footnotes

1
https://seedglobalhealth.org/
